# *In-situ* Real-Time Monitoring of Volatile Organic Compound Exposure and Heart Rate Variability for Patients with Multiple Chemical Sensitivity

**DOI:** 10.3390/ijerph121012446

**Published:** 2015-10-05

**Authors:** Atsushi Mizukoshi, Kazukiyo Kumagai, Naomichi Yamamoto, Miyuki Noguchi, Kazuhiro Yoshiuchi, Hiroaki Kumano, Kou Sakabe, Yukio Yanagisawa

**Affiliations:** 1 Department of Environmental Medicine and Behavioral Science, Kinki University Faculty of Medicine, 377-2, Ohno-higashi, Osakasayama, Osaka 589-8511, Japan; 2 Institute of Applied Brain Sciences, Waseda University, 2-579-15 Mikajima, Tokorozawa, Saitama 359-1192, Japan; E-Mail: hikumano@waseda.jp; 3 Environmental Health Laboratory, California Department of Public Health, 850 Marina Bay Pkwy, Richmond, CA 94804, USA; E-Mail: kkumagai@cdph.ca.gov; 4 Department of Environmental Health Sciences, Graduate School of Public Health, Seoul National University, 1 Gwanak-ro, Gwanak-gu, Seoul 151-742, Korea; E-Mail: nyamamoto@snu.ac.kr; 5 Department of Materials and Life Science, Faculty of Science and Technology, Seikei University, 3-3-1 Kichijoji-kitamachi, Musashino, Tokyo 180-8633, Japan; E-Mail: noguchi@ejs.seikei.ac.jp; 6 Department of Stress Sciences and Psychosomatic Medicine, Graduate School of Medicine, The University of Tokyo, Hongo 7-3-1, Bunkyo-ku, Tokyo 113-8655, Japan; E-Mail: kyoshiuc-tky@umin.ac.jp; 7 Faculty of Human Sciences, Waseda University, 2-579-15 Mikajima, Tokorozawa, Saitama 359-1192, Japan; 8 Department of Anatomy and Cellular Biology, Tokai University School of Medicine, 143 Shimokasuya, Isehara, Kanagawa 259-1193, Japan; E-Mail: sakabek@tokai-u.jp; 9 The University of Tokyo, 7-3-1, Hongo, Bunkyo-ku, Tokyo 113-8654, Japan; E-Mail: yukio@kaiseigakuen.jp

**Keywords:** real-time monitoring, MCS, VOC, HRV

## Abstract

*In-situ* real-time monitoring of volatile organic compound (VOC) exposure and heart rate variability (HRV) were conducted for eight multiple chemical sensitivity (MCS) patients using a VOC monitor, a Holter monitor, and a time-activity questionnaire for 24 h to identify the relationship between VOC exposure, biological effects, and subjective symptoms in actual life. The results revealed no significantly different parameters for averaged values such as VOC concentration, HF (high frequency), and LF (low frequency) to HF ratio compared with previous data from healthy subjects (*Int. J. Environ. Res. Public Health* **2010**, *7*, 4127–4138). Significant negative correlations for four subjects were observed between HF and amounts of VOC change. These results suggest that some patients show inhibition of parasympathetic activities along with VOC exposure as observed in healthy subjects. Comparing the parameters during subjective symptoms and normal condition, VOC concentration and/or VOC change were high except for one subject. HF values were low for five subjects during subjective symptoms. Examining the time-series data for VOC exposure and HF of each subject showed that the subjective symptoms, VOC exposure, and HF seemed well related in some symptoms. Based on these characteristics, prevention measures of symptoms for each subject may be proposed.

## 1. Introduction

Multiple chemical sensitivity (MCS) has been defined as an acquired disorder characterized by recurrent symptoms, referable to multiple organ systems, occurring in response to demonstrable exposure to many chemically unrelated compounds at doses far below those established in the general population to cause harmful effects [1]. Medical researchers and clinicians from the United States and Canada signed the 1999 Consensus on MCS and established the following criteria: (1) The symptoms are reproducible with (repeated chemical) exposure; (2) The condition is chronic; (3) Low levels of exposure (lower than previously or commonly tolerated) result in manifestations of the syndrome; (4) The symptoms improve or resolve when the incitants are removed; (5) Responses occur to multiple chemically unrelated substances; (6) Symptoms involve multiple organ systems [2].

As defined above, chemical exposure has been assumed to trigger the symptoms, although the underlying mechanism of MCS remains disputed. Therefore, it is inevitable to investigate the relationship between chemical exposure and symptoms for understanding the pathogenesis, making a diagnosis, and proposing a measure of cure and prevention of symptoms. Provocation challenges have been conducted to clarify whether the patients actually show different responses to low levels of exposure [3]. These tests are conducted in controlled environments. However, in actual life where various chemicals exist, it is assumed that “masking” (acclimatization, apposition, and addiction) may hide the exposure-symptom relationships [4]. As a result, exposure-symptom relationships in actual lives may be different from those in controlled environments. It is therefore essential to understand the actual conditions of MCS measurements of chemical exposure and symptoms in actual life. Few studies have been done measuring chemical exposure of patients in actual lives and evaluating the relationship to the symptoms. Shinohara *et al.* [5] measured the exposures of 15 MCS patients to both carbonyl compounds and volatile organic compounds (VOCs) that may induce hypersensitive reaction in actual lives. The results demonstrated that the chemicals responsible for hypersensitive reactions varied from patient to patient. Moreover, the concentration during symptoms was far below the WHO indoor guidelines. Saito *et al.* [6] used Ecological Momentary Assessment to monitor everyday symptoms in addition to the environmental chemical exposure measurement. The results showed that some causative chemicals were detected in 11 of 14 MCS patients and 11 physical symptoms and four mood subscales were significantly aggravated when they experienced hypersensitivity symptoms. In those studies, chemical compounds were collected by adsorbents and the composition and concentration were analyzed using gas chromatography-mass spectrometry (GCMS) and high-performance liquid chromatography (HPLC). These integration analysis tests are general methods to measure the concentration of volatile organic compound (VOC) components and the result obtained by this method is the average concentration during a certain period (e.g., from 30 min to 1 week).

To elucidate the responsible exposure for each symptom, it is necessary to detect the fluctuation of personal exposure because personal exposure fluctuates according to personal activities and locations in a short period. However, it is difficult to obtain the personal exposure fluctuation from the data of average concentration. On the other hand, measurement methods with higher time resolution such as real-time monitoring using portable VOC monitors to measure total VOC concentrations [7,8,9] provide time-series data of personal exposure concentrations, but do not elucidate the VOC components. Therefore, it is assumed that using these monitors the fluctuation of personal exposure can be detected and the information about the context or simultaneity between exposure and symptoms of MCS patients can be obtained.

To investigate the relationship between the fluctuation of VOC exposure and its biological effects, it is necessary to know the change in biological parameters in a relatively short time. Since MCS patients usually report various autonomic nerve symptoms, it is desirable to know the temporal changes in autonomic nerve function in actual life. Heart rate variability (HRV) measured by Holter monitor has been used to evaluate the biological effects caused by environmental factors [10,11,12,13,14,15,16,17].

In our previous study, VOC exposure concentrations and HRV using VOC and Holter monitors were measured for seven healthy subjects [18]. In this study, we applied this method to MCS patients and identified characteristics of the relationship between VOC exposure, biological effects, and subjective symptoms in actual lives. In this paper, first, VOC exposure and HRV parameters of MCS patients were compared to controls. Moreover, the correlations between VOC exposure and HRV parameters were considered. Further, the parameters during subjective symptom and normal condition were compared. Finally, time-series data for each subject were observed in detail.

## 2. Experimental Section

### 2.1. Study Design

This study was designed to simultaneously monitor personal VOC concentrations and HRV for eight MCS patients under usual daily life conditions. The measurements were conducted from 2006 to 2007. The subjects were requested to wear the Holter monitor, carry the VOC monitor, and record the time-activity logs during monitoring.

All subjects gave their informed consent for inclusion before they participated in the study. The study was conducted in accordance with the Declaration of Helsinki, and the protocol was approved by the Research Ethical Committee of the Kitasato Institute Hospital of No.13 D-180-10.

### 2.2. Subjects

The subjects were eight MCS patients including three adult males and five adult females, and the ages ranged from 31 to 62 years (44 ± 11 years). These patients consulted doctors in the Division of Environmental Medical Center, Kitasato Institute Hospital. Various examinations for diagnosis, including neuro-ophthalmologic examination, medical examination by interview, and questionnaire survey were performed in a clean room. The questionnaire included the reason for visiting, subjective symptoms, questions about life environment, and the Quick Environment Exposure Sensitivity Inventory (QEESI) in Japanese [19]. The patients were diagnosed with MCS by medical specialists from the comprehensive results of these examinations.

Because the responsible chemical compounds are expected to be different for each patient, it is difficult to lump MCS patients together. Therefore, MCS patients were limited to those advanced from Sick Building Syndrome whose responsible compounds were considered to be VOCs.

### 2.3. VOC Monitoring

VOC exposure concentrations were measured for 24 h by a portable real-time VOC monitor with photo ionization detector (PID) (ppbRAE plus; RAE Systems, San Jose, CA, USA). Detailed information on VOC monitoring has been described in a previous study [18]. Before each measurement, calibrations were conducted using 10 or 100 ppm isobutylene gas. Temperature and relative humidity (RH) were measured by a thermo-hygrometer (HOBO; Onset Computer Corporation, Jackson, MS, USA) carried with the VOC monitor.

For the analysis, VOC concentration averaged over each 5-min interval and the changes in VOC concentration amount in 5-min interval (ΔVOC), calculated by subtracting the minimum value from the maximum value of each interval, were used. Additionally, the differential changes of 5-min averaged VOC concentration from the previous 5-min averaged VOC concentration were calculated and divided into positive values (d + VOC) and negative values (d − VOC). Temperature and RH, averaged over each 5-min interval, were also used for the analysis.

### 2.4. HRV Analysis

The continuous electrocardiogram (ECG) data were recorded for 24 h by the Holter monitor (FM-150 or FM-180; Fukuda Denshi, Tokyo, Japan). Detailed information on HRV analysis has been described in a previous study [18]. To avoid eliciting a response to the electrode seals, they were exposed to air before use and dispelled their smell as much as possible.

For the analysis, high frequency (HF) and low frequency (LF) power were averaged over 5-min intervals, HF power was used as an indicator of parasympathetic activity, and the power ratio of 5-min averaged LF to 5-min averaged HF (LF/HF) was used as an indicator of sympathetic activity [20,21].

### 2.5. Time-Activity Pattern

**Figure 1:**
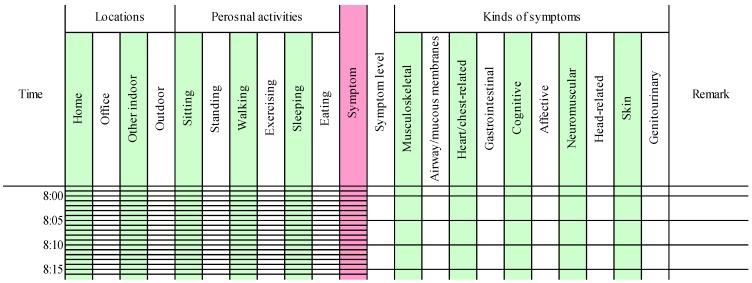
An example of a time-activity log sheet.

The time-activity patterns were recorded by the subjects (Figure 1). The subjects were requested to select their locations and activities in each 5-min interval from the following alternatives: four kinds of locations including home, office, other indoor, and outdoor; and six kinds of personal activities including sitting, standing, walking, exercising, eating, and sleeping. In addition, when a symptom was induced, subjects were instructed to indicate the symptom level on a 0–10 scale (0 = not at all a problem, 5 = moderate symptoms, and 10 = disabling symptoms) and select the type from the symptom severity items in QEESI [22,23] including musculoskeletal, airway/mucous membranes, heart/chest-related, gastrointestinal, cognitive, affective, neuromuscular, head-related, skin, and genitourinary. Responsible exposure chemicals or events could be written in the remarks column.

To ignore the confounding factors, we excluded the data of the time spent for these activities (*i.e.*, exercising, eating, and sleeping) and the duration of the effect (*i.e.*, 15 min after exercising and 1 h after eating) from the analyses in the same manner as the previous study [18].

### 2.6 Statistical Analysis

VOC exposure concentration and HRV parameters of MCS patients were compared to controls using Wilcoxon non-parametric test. To assess the relationships between VOC exposures and HRV parameters Spearman rank correlation coefficients were calculated. Time ratios of respective symptoms were analyzed using principal component analysis to figure out the characteristics of subjective symptoms. The parameters during subjective symptom and normal condition were compared using Wilcoxon non-parametric test. All analysis were conducted using IBM SPSS Statistics Version 22 (IBM).

## 3. Results

### 3.1. Statistical Summary

Table 1 shows a summary of VOC exposure concentrations and HRV parameters observed for all subjects who participated in this study. The results indicate that exposure concentrations differed for each patient. Table 1 also shows control (healthy subject). Only the data from a previous study [18] was used. No significant difference was observed between patients and healthy subject for all the parameters (Wilcoxon non-parametric test). 

**Table 1:** Summary of VOC exposure concentrations and HRV parameters for patients and controls.

Parameters	Patients		Controls ^c^		*p* ^d^
	n ^a^	Mean ± SD ^b^	n	Mean ± SD	
VOC exposure concentration (µg·m^−3^)					
Total	8	306 ± 148	7	176 ± 130	0.12
Home	8	262 ± 204	7	299 ± 267	1.00
HRV parameters					
Log_10_HF (m·sec^2^)	8	1.5 ± 0.2	7	1.7 ± 0.4	0.34
LF/HF	8	3.8 ± 1.9	7	3.8 ± 2.2 ^e^	1.00

**^a^** Sample size; **^b^** Standard deviation; **^c^** Data from a previous study [18]; **^d^** Wilcoxon non-parametric test; **^e^** LF/HF was calculated using LF and HF averaged over 5-min intervals.

### 3.2. Bivariate Analysis

Spearman rank correlation coefficients were calculated to assess the relationships between VOC exposures and HRV parameters measured within the same 5-min intervals. Table 2 shows a summary of the correlations for all subjects. The sex and age of these subjects are also listed in Table 2.

**Table 2:** Correlations between VOC exposure and HRV parameters.

Parameters	Subject								− ^d^	+ ^e^
	A	B	C	D	E	F	G	H		
Sex (M: male, F: female)	M	F	F	F	M	M	F	F		
Age (years)	39	62	33	46	31	49	35	54		
VOC *vs.* HF	−0.35 ** ^a^	0.19 *	−0.03	−0.00	−0.34 **	−0.04	0.06	−0.13	6 (2)	2 (1)
ΔVOC *vs.* HF	−0.38 **	0.01	0.11	−0.32 **	0.07	−0.03	−0.24 *	−0.30 **	5 (4)	3 (0)
d+VOC *vs.* HF	−0.46 **	−0.17	0.13	−0.37 *	0.02	0.04	−0.38 *	−0.45 **	5 (4)	3 (0)
d−VOC *vs.* HF	0.31 * ^b^	−0.08	0.09	0.45 **	0.30 **	0.39 **	0.15	0.34 **	1 (0)	7 (5)
VOC *vs.* LF/HF	0.06	−0.22 *	0.01	−0.16	0.26 **	−0.04	−0.06	0.01	4 (1)	4 (1)
ΔVOC *vs.* LF/HF	0.08	0.11	0.02	0.28 **	−0.09	−0.08	0.29 **	0.04	2 (0)	6 (2)
d+VOC *vs.* LF/HF	0.04	0.10	0.27	0.45 **	0.04	−0.18	0.27	0.12	1 (0)	7 (1)
d−VOC *vs.* LF/HF	0.13	−0.21	−0.13	−0.33 *	−0.27 **	−0.30 *	−0.21	−0.08	7 (3)	1 (0)
Temp *vs.* HF	−0.01	0.38 **	−0.07	0.13	- ^c^	0.15	0.21 *	−0.40 **	3 (1)	4 (2)
RH *vs.* HF	0.10	−0.61 **	0.15	0.18	-	−0.21 *	0.16	0.38 **	2 (2)	5 (1)
Temp *vs.* LF/HF	0.20 *	−0.12	−0.02	−0.03	-	−0.04	−0.12	0.09	5 (0)	2 (1)
RH *vs.* LF/HF	−0.08	−0.15	−0.14	−0.22 *	-	0.18	−0.20 *	−0.10	6 (2)	1 (0)

^a^ ** Spearman rank correlation, *p* < 0.01; ^b^ * Spearman rank correlation, *p* < 0.05; ^c^ Data not obtained; ^d^ − Numbers of the subjects showing negative correlation (significant); ^e^ + Numbers of the subjects showing positive correlation (significant).

### 3.3. Characteristics of Subjective Symptoms

**Figure 2:**
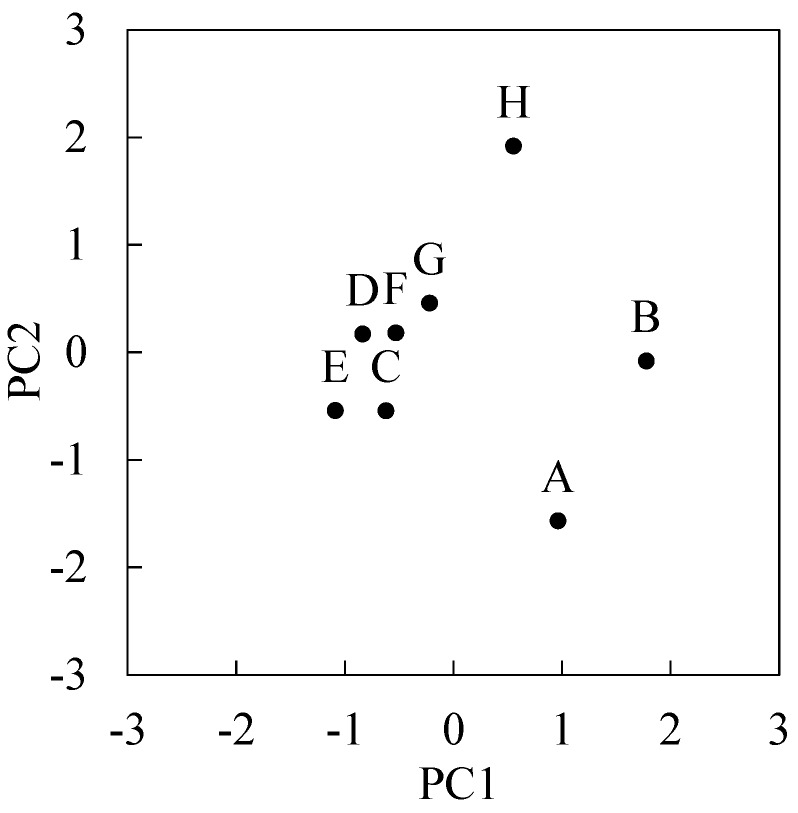
Plot of first principal component (PC1) and second principal component (PC2) scores of eight subjects.

Table 3 shows subjective symptom times and time ratios of respective symptoms for all subjects. These counted all symptom times in a 5-min interval including those during exercising, eating, and sleeping. Average time of symptom was 3.2 ± 3.0 (0.3–9.8) h. The order of frequency of symptoms is airway/mucous membranes (8/8 subjects), heart/chest-related (6/8 subjects), gastrointestinal (5/8 subjects), cognitive (4/8 subjects), neuromuscular (4/8 subjects), head-related (4/8 subjects), musculoskeletal (3/8 subjects), affective (3/8 subjects), skin (2/8 subjects), and genitourinary (1/8 subjects). Principal component analysis was performed on the variance-covariance matrix (8 subjects × 10 variables), where the variables comprised the time ratios of respective symptoms. Figure 2 shows principal component 1 (PC1) and principal component 2 (PC2) scores for each subject, which accounted for 78% variance. This result indicates the characteristics of symptoms of subject A, B, and H were different from other subjects.

**Table 3:** Subjective symptom time (h) and time ratio of respective symptoms (%).

Symptoms	Subject							
	A	B	C	D	E	F	G	H
Symptom time	2.7	9.8	4.8	0.8	2.8	0.3	2.4	2.1
Musculoskeletal	0	8	0	0	52	0	0	4
Airway/mucous membranes	94	84	7	22	3	75	97	84
Heart/chest-related	0	84	24	44	0	25	17	92
Gastrointestinal	0	5	40	22	3	0	0	4
Cognitive	0	81	5	0	0	0	31	84
Affective	0	1	2	0	0	0	0	4
Neuromuscular	91	92	10	0	0	0	0	12
Head-related	94	92	43	0	18	0	0	0
Skin	0	2	0	0	0	0	0	64
Genitourinary	0	0	0	0	0	0	7	0

### 3.4. Comparison between Subjective Symptom Time and Normal Time

Table 4 compares subjective symptom times and normal condition times. Because the symptom of subject F was observed for only one interval (5-min) after excluding confounding factors, the data is eliminated.

**Table 4:** Comparison of the parameter of subjective symptom time and normal condition time.

Parameters	Subject							↓ ^f^	↑ ^g^
	A	B	C	D	E	G	H		
VOC	↓ ^a^	↑ **	↓	↑	↑ *	↑	↑	2 (0)	5 (2)
ΔVOC	↑ ^b^	↑	↑	↓	↑ *	↑	↓	2 (0)	5 (1)
HF	↓ ** ^c^	↓ **	↓ **	↓	↑ **	↑	↓	5 (3)	2 (1)
LF/HF	↑ * ^d^	↓	↑ **	↑	↓	↓	↓	4 (0)	3 (2)
Temp	↓	↓	↑ *	↓	- ^e^	↓ **	↓ **	5 (2)	1 (1)
RH	↑ **	↑ **	↑	↑	-	↑ *	↑ *	0 (0)	6 (4)

^a^ ↓ Average value was lower during symptoms; ^b^ ↑ Average value was higher during symptoms; ^c^ ** Wilcoxon non-parametric test, *p* < 0.01; ^d^ * Wilcoxon non-parametric test, *p* < 0.05; ^e^ Data not obtained; ^f^ Number of the subjects showing lower during symptoms (significant); ^g^ Number of the subjects showing higher during symptoms (significant).

## 4. Discussion

### 4.1. Comparisons of VOC Exposure and HRV Parameters between Patients and Controls

It is assumed that patients avoid exposure to chemicals, and consequently their exposure level of chemical compounds is lower than that of healthy subjects [5]. However, the patients’ VOC exposure concentration in total was higher than that of controls in this study although the differences were not significant (Table 1). The VOC concentration that were measured by the VOC monitor used in this study included various VOCs. Thus, chemicals that did not induce symptoms in patients may have been included in the measurements, which could explain why the VOC exposure concentrations of patients were not lower than those in healthy subjects. In addition, it should be taken into consideration that the subjects were recruited from volunteers and were not representative of a random sample of controls or MCS patients. With regard to HRV parameters, HF of patients had a low tendency although the differences were not significant.

### 4.2. Correlations between VOC Exposure and HRV Parameters

Significant negative correlation in two out of eight subjects and significant positive correlation in one out of eight subjects were observed between VOC concentration and HF (Table 2), suggesting there is no consistent trend between VOC concentration and HF for MCS patients. On the other hand, significant negative correlations between ΔVOC and HF were observed in four out of eight subjects, suggesting that changes of VOC concentrations in 5-min intervals were associated with decreased activity of parasympathetic nervous system. In addition, significant negative correlations between d + VOC and HF were observed in four out of eight subjects, and significant positive correlations between d − VOC and HF were seen in five out of eight subjects. These results indicate that HF tends to be low when the VOC concentrations increase or decrease. If VOC exposure is the cause of decreased HF, it is assumed that decreased VOC exposure enhances HF power. However, HF was low when the VOC concentration decreased. Based on the existence of a causal relationship between exposure and the parasympathetic nervous system, one possibility is to assume that decrease of VOC exposure as with increase causes the HF decrease. Another possibility is that a delay of HF increases after VOC exposure. Between LF/HF and ΔVOC, significant positive correlation was observed in two out of eight subjects. Significant positive correlations were observed between d + VOC and LF/HF in one out of eight subjects, and significant negative correlations were observed between d − VOC and LF/HF in three out of eight subjects. These tendencies were opposite to HF.

As in the previous study, negative correlations between ΔVOC and HF in six out of seven healthy subjects were observed. Since these tendencies were more frequent in healthy subjects than in patients, there is a possibility that the absence of significant correlation between ΔVOC and HRV parameters is characteristic of MCS patients.

### 4.3. Comparison between Subjective Symptom Time and Normal Time

During subjective symptoms compared to normal conditions, VOC concentration was higher in five subjects and ΔVOC was higher in five subjects [significant differences were observed in subject B and E (Wilcoxon non-parametric test)] (Table 4). That is, VOC concentration and/or the change amount were high in all subjects except for subject F. In relation to HRV, the values of HF in five subjects were low during subjective symptoms (significant differences were observed in three subjects). These tendencies suggested the presence of high VOC concentration or change and low HF power when the subjects feel symptoms. In addition, the RH was high during subjective symptoms in six subjects, suggesting that RH has some relationship to subjective symptoms.

### 4.4. Case Studies

The measurement in this study was not designed to clarify the causal linkage between exposure and symptoms, but the context or simultaneity between exposure and symptoms can be observed from the time-series data. Therefore, time-series data for each subject were observed in detail in each case. To grasp the tendencies visually, average and maximum VOC concentration during 1 min and log_10_ HF during 1 min and 15-min moving average of log_10_ HF are indicated. Here it was assumed that the symptoms occurred when the subject sensed the exposure of chemicals. The longtime delay of symptom occurrence after exposure was not considered. This is based on the survey which clarified that the timing of symptom occurrence was almost immediately after an exposure [24].

Moreover, from this information, preventive measures were proposed for each subject. There is no common MCS treatment protocol accepted across medical disciplines. Gibson *et al.* surveyed perceived treatment efficacy for conventional and alternative therapies reported by a person with MCS. As a result, participants rated chemical avoidance, creating a chemical-free living space, and prayer as the three most useful interventions [25]. On the other hand, cognitive therapy, such as mindfulness, are being explored as treatment option for MCS [26,27].

This study includes several limitations attributed to various confounding factors based on the measurements in actual lives. VOC monitors measure the concentration of a wide variety of environmental VOCs in total, including non-symptom-related VOCs. Moreover, HRV parameters are affected by various environmental factors and personal activities. In addition, the relationship between exposure and symptoms cannot refer to causal relationship. However, this method provided numerically-expressed data for actual condition of MCS exposure and symptoms which had been assumed depending on the interview and suggested a new insight into treatment processes.

#### 4.4.1. Subject A

**Figure 3:**
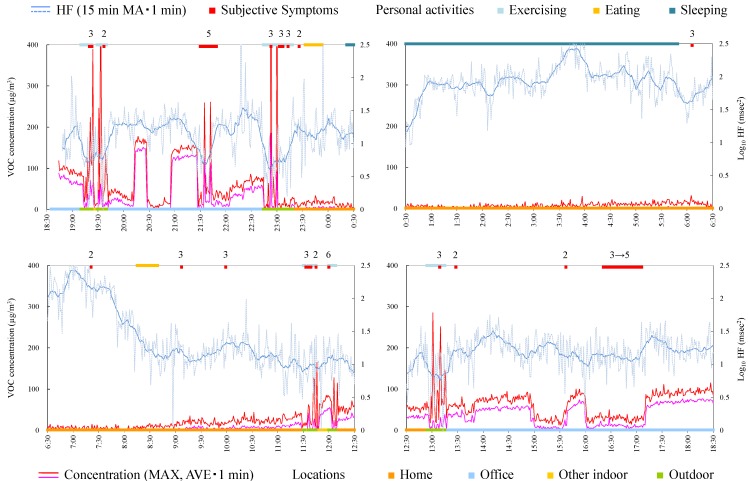
Time-series data of VOC concentration and log_10_ HF (subject A). Subjective symptoms and personal activities are indicated in upper side and locations are indicated in lower side. The values on subjective symptoms are symptom levels.

Subject A was a 39-year-old male and a researcher. Responsible exposures during monitoring were insecticides, tobacco, cosmetics, refresher, paint, detergent, smoke, and disinfectants inside a building. Figure 3 shows time-series data of VOC concentration and log_10_ HF of subject A. In the time from 18:30 to 0:30, several peak shape VOC concentration changes were observed and almost simultaneously (just before or immediately after the peak exposure) the subject felt the symptoms. This indicates that symptoms were induced when he was exposed to some VOCs (although symptoms before acute exposure may be anticipatory symptoms). It is noteworthy that log_10_ HF decreased before the occurrence of symptoms during exposure to relatively high and successive concentrations indoors (e.g., 21:00–21:30). From these findings, the exposure and symptom relationship is assumed as below: when HF is decreasing, a symptom is induced along with sudden elevation of VOC concentration. Meanwhile, a symptom occurred without high concentration of VOC, for example, during 16:20–17:10.

Consequently, since the symptom of this subject seemed synchronized with acute exposure, the measure to prevent symptoms for this subject is to avoid the condition or environment where the acute exposure of chemical or the environment (particularly outdoors) occurs. In addition, enhancing the parasympathetic nerve activities in daily life may be effective.

#### 4.4.2. Subject B

**Figure 4:**
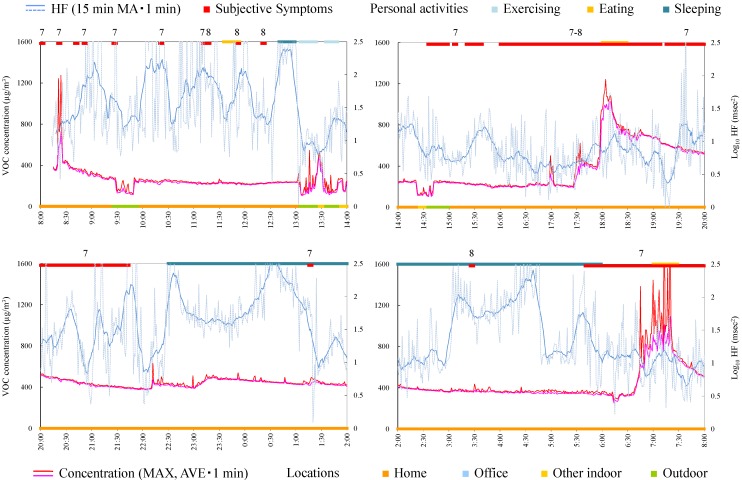
Time-series data of VOC concentration and log_10_ HF (subject B). Subjective symptoms and personal activities are indicated in upper side and locations are indicated in lower side. The values on subjective symptoms are symptom levels.

Subject B was a 62-year-old female and a homemaker. Figure 4 shows time-series data of VOC concentration and log_10_ HF of subject B. During 8:00–8:30, a peak exposure was observed and at the same time symptoms were induced. However, the symptoms occurred frequently during 8:30–13:00, although the concentration of VOC hardly fluctuated. Therefore, in this case it was suggested that the subject mostly felt symptoms without increased VOC concentration. This suggests that the VOC monitor could not measure the concentration or concentration change of the chemicals that induced symptoms in this subject. For example the VOC monitor used in this study cannot detect formaldehyde which was rated as causing most symptomatology in persons self-identified with MCS [28]. Alternatively, this suggests that the subject repeated learned symptoms that could have been induced by a similar environment or condition in which symptoms were previously provoked by specific chemicals. HF during symptoms was significantly lower than that during normal conditions (Table 4). From this standpoint, other measures except for avoiding exposure, such as neurological treatment or cognitive therapy, may be effective.

#### 4.4.3. Subject C

**Figure 5:**
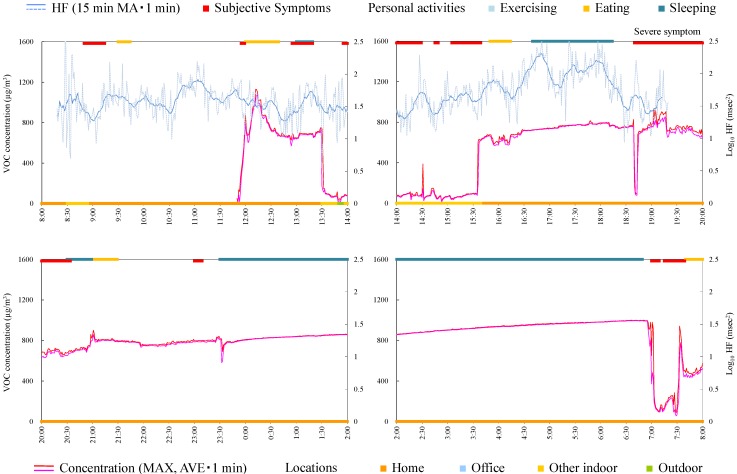
Time-series data of VOC concentration and log_10_ HF (subject C). Subjective symptoms and personal activities are indicated in upper side and locations are indicated in lower side. The values on subjective symptoms are symptom levels.

Subject C was a 33-year-old female and a homemaker. Since symptoms worsened during measurement, the Holter monitor was removed at 19:30. Responsible exposures during monitoring were car exhaust, open burning, pesticide, tobacco, personal computer, and stove. Figure 5 shows time-series data of VOC concentration and log_10_ HF of subject C. The concentration in the house was relatively higher than that in other environments because the subject lived in new residential housing. In fact, measurement of VOCs in breath in the clean room indicated a high concentration of α-pinene, suggesting that α-pinene emitted from new wood material was absorbed by the body. Since the symptoms were not always induced in the house, it was suggested that VOCs existing on a steady basis in the house, including α-pinene, were not possible compounds for this subject. At 14:45 a symptom was induced in a car by open burning and almost simultaneously increase in VOC concentration was detected. Severe symptom was induced at 18:40 when a guest came. The concentration of VOC rapidly decreased and increased. This may be because of incursion of outdoor air by opening the front door. Because VOC concentration of outdoor air was low compared with indoor air, the concentration increase by responsible compounds could not be detected. This is a limitation of the measurement using the VOC monitor, which cannot separate the components.

HF during symptoms was significantly lower than during normal condition (Table 4) and some symptoms seem to be induced after HF decreases. Therefore, taking care to live a life enhancing the parasympathetic nerve activity may be effective also for this subject.

#### 4.4.4. Subject D

**Figure 6:**
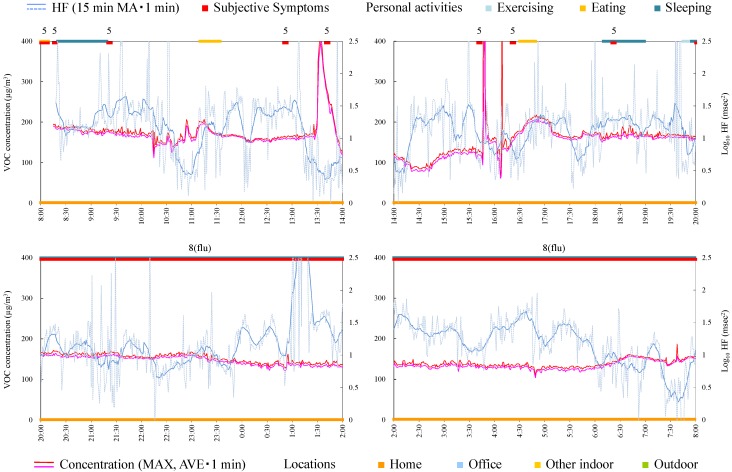
Time-series data of VOC concentration and log_10_ HF (subject D). Subjective symptoms and personal activities are indicated in upper side and locations are indicated in lower side. The values on subjective symptoms are symptom levels.

Subject D was a 46-year-old female and a homemaker. Figure 6 shows time-series data of VOC concentration and log_10_ HF of subject D. After 20:00, the subject developed symptoms of flu. As is the case with subject A, sets of tendencies (after HF decrease, the subjective symptom, and acute increase of VOC concentration) were observed during 13:30–14:00 and 15:30–16:30. Therefore, avoiding the condition or environment of acute exposure and enhancing the parasympathetic nerve activities may be effective for prevention of symptoms.

#### 4.4.5. Subject E

**Figure 7:**
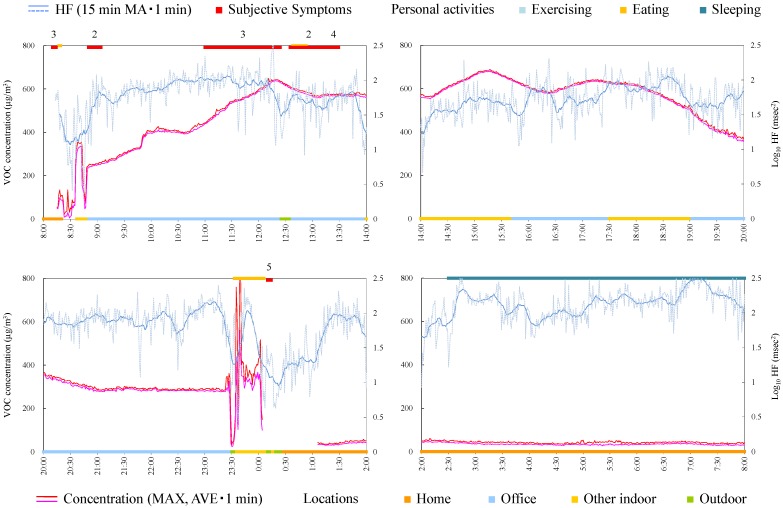
Time-series data of VOC concentration and log_10_ HF (subject E). Subjective symptoms and personal activities are indicated in upper side and locations are indicated in lower side. The values on subjective symptoms are symptom levels.

Subject E was a 31-year-old male and an office worker. Figure 7 shows time-series data of VOC concentration and log_10_ HF of subject E. HF was significantly high during symptoms (Table 4). The symptom from 11:00 was induced while the VOC concentration was increased in office. Therefore, creating a chemical-free living space may be necessary.

#### 4.4.6. Subject F

**Figure 8:**
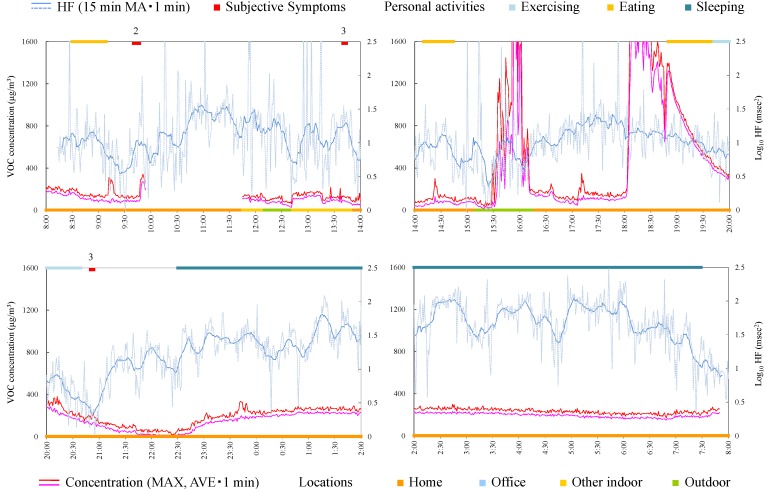
Time-series data of VOC concentration and log_10_ HF (subject F). Subjective symptoms and personal activities are indicated in upper side and locations are indicated in lower side. The values on subjective symptoms are symptom levels.

Subject F was a 49-year-old male and an office worker. Responsible exposures during monitoring were liquid detergents used for cleaning and tobacco. Figure 8 shows time-series data of VOC concentrations and log_10_ HF of subject F. The symptoms were induced by relatively low concentrations at 9:40 and 13:40. After HF was decreased by exercise, the subjective symptom appeared during slight change of VOC concentration at 20:45. Meanwhile, symptoms were not induced by VOC concentrations by large increase and fluctuation from 15:30 and from 18:00. Therefore, avoiding the responsible exposures, such as detergents and tobacco, may be effective to prevent the symptoms for this subject.

#### 4.4.7. Subject G

**Figure 9:**
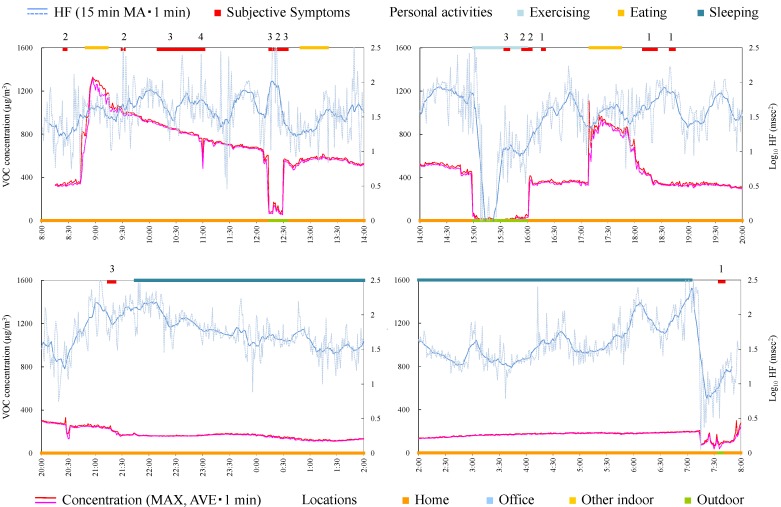
Time-series data of VOC concentration and log_10_ HF (subject G). Subjective symptoms and personal activities are indicated in upper side and locations are indicated in lower side. The values on subjective symptoms are symptom levels.

Subject G was a 35-year-old female and on leave from her job. Responsible exposures during monitoring were odor of new organic cotton, bag for measurement apparatus, print, paper, pencil, ballpoint pen, envelope, odor of detergents, odor of drugstore, odors of cosmetics, perfume, and tobacco, odors of clothes and hair dressing, exhaust gas, smoke, fragrance, and odors of the dryer and closet. Figure 9 shows time-series data of VOC concentration and log_10_ HF of subject G. VOC concentration was very high during daytime, in the home, after breakfast (8:50). Consistent tendencies were not observed between VOC exposure, symptoms, and HF. Because the symptoms in this subject were mainly induced by odors of consumer products, which may affect only a local space and may not reach to the monitor sufficiently, there was a possibility that the monitor could not detect their effects.

**Figure 10:**
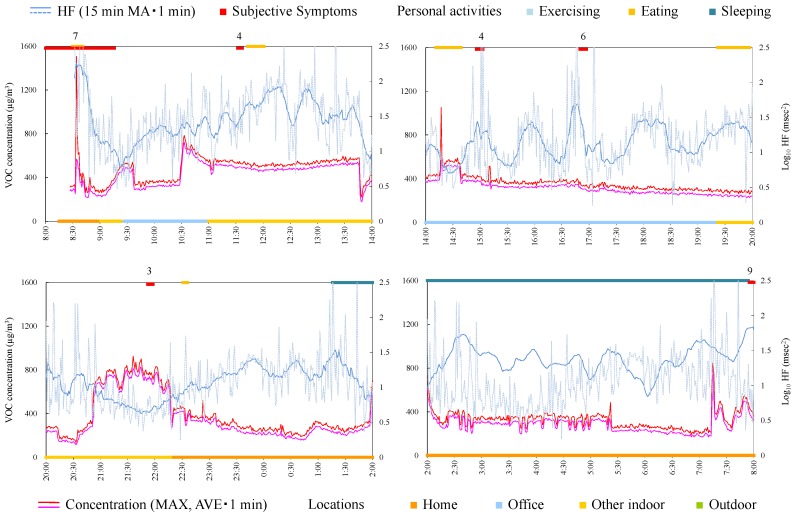
Time-series data of VOC concentration and log_10_ HF (subject H). Subjective symptoms and personal activities are indicated in upper side and locations are indicated in lower side. The values on subjective symptoms are symptom levels.

#### 4.4.8. Subject H

Subject H was a 54-year-old female and a company owner. Figure 10 shows time-series data of VOC concentration and log_10_ HF of subject H. After decrease in HF, the subjective symptom appeared during relatively high concentration at 21:55. For other symptoms, consistent tendencies were not observed between VOC exposure, symptoms, and HF.

## 5. Conclusions

*In-situ* real-time monitoring of VOC exposure and HRV were conducted for eight MCS patients using a VOC monitor, a Holter monitor, and a time-activity pattern for 24 h to identify the relationship between VOC exposure, biological effects, and subjective symptoms of MCS patients in actual life. The results showed that there were no significantly different parameters for averaged values such as VOC exposure concentration, HF, and LF/HF compared with previous data from healthy subjects. Between HF and VOC change amount, significant negative correlations for four out of eight subjects were observed. These results suggest that some patients show inhibition of parasympathetic activities along with VOC exposure as seen with healthy subjects in a previous study. Comparing the parameters during subjective symptoms and normal conditions, VOC concentrations and/or VOC change amounts were high in all subjects except for one, and the values of HF were low for five subjects during subjective symptoms, suggesting the presence of high VOC concentration or change and low HF power when the subjects feel symptoms. Examining the time-series data for VOC exposure and log_10_ HF of each subject revealed subjects whose subjective symptoms, VOC exposure, and HF seemed well related and other subjects whose findings did not appear related. Though there are limitations of the study design and the method, characteristics of relationship between exposure and symptoms were suggestive, and based on these characteristics, prevention measures of symptoms for each subject may be proposed.
